# 多发性骨髓瘤相关静脉血栓栓塞症防治中国专家共识（2022年版）

**DOI:** 10.3760/cma.j.issn.0253-2727.2022.09.003

**Published:** 2022-09

**Authors:** 中华医学会血液学分会浆细胞疾病学组 中华医学会血液学分会血栓与止血学组

多发性骨髓瘤（multiple myeloma, MM）是一种浆细胞克隆性疾病, 由于大多数患者为高龄且伴有高免疫球蛋白血症所致高凝状态、应用免疫调节剂（IMiD）和大剂量糖皮质激素以及因手术或疼痛制动等危险因素，容易并发静脉血栓栓塞症（venous thromboembolism, VTE）。VTE的发生不仅限制了治疗药物的应用，还严重影响患者的生活质量、致残甚至威胁生命。为了规范国内MM的治疗、降低患者VTE风险，中华医学会血液学分会浆细胞疾病学组、血栓与止血学组联合召集国内相关领域专家，基于不同级别循证医学依据制定本版中国MM相关VTE防治专家共识。

一、MM相关VTE的临床表现

MM相关VTE的首发表现多为深静脉血栓（deep venous thrombosis, DVT），也可以表现为肺栓塞（pulmonary embolism, PE）。DVT以下肢多见，约占所有DVT的80％[1]–[2]，主要表现为不同程度的肢体疼痛、水肿、触痛以及浅静脉曲张等。PE主要表现为不同程度的呼吸困难、胸痛、咯血、心动过速和低氧血症，严重者可表现为严重的血流动力学障碍（低血压、休克、晕厥、神志不清、猝死等）。

二、MM相关VTE的诊断

MM的诊断主要参考《中国多发性骨髓瘤诊治指南（2022年修订）》[3]。VTE的诊断需综合临床表现或临床评分并结合D-二聚体定量分析和相关的超声检查或增强CT、MRI进行评价，必要时采用血管造影术进行明确或排除。DVT、PE的临床评分建议采用Wells评分系统[4]–[5]（表1、表2），据此作出VTE可能性高低的临床判断。对疑似新发的DVT或PE患者，若D-二聚体结果正常则有助于排除诊断。

**表1:** 深静脉血栓（DVT）Wells评分[4]

因素	积分
活动性肿瘤	+1
下肢瘫痪或近期石膏固定	+1
制动大于3 d或近1个月内拟行大型手术	+1
沿深静脉走行局部压痛	+1
全腿肿胀	+1
小腿肿胀大于3 cm（与无症状侧相比）	+1
单侧凹陷性水肿	+1
浅静脉侧支循环	+1
既往有深静脉血栓史	+1
临床表现类似DVT的其他诊断	−2

注：临床可能性：不可能：总分≤2分（发生率4％）；有可能：总分>2分（发生率27％）

**表2:** 肺栓塞（PE）Wells评分[5]

因素	积分
其他诊断的可能性低于PE	+3
有深静脉血栓（DVT）症状和体征	+3
心率>100次/min	+1.5
既往静脉血栓栓塞症（VTE）病史	+1.5
4周内制动或手术	+1.5
活动性肿瘤	+1
咯血	+1

注：临床可能性低：总分≤1分（发生率4％）；临床可能性中：总分2～6分（发生率21％）；临床可能性高：总分>6分（发生率67％）；不可能：总分≤4分（发生率8％）；可能：总分>4分（发生率41％）

三、MM相关VTE的鉴别诊断

临床上疑似DVT的患者需要与肌肉扭伤或断裂、下肢扭伤、淋巴管炎或淋巴管梗阻、静脉逆流、腘窝囊肿、蜂窝织炎、肾功能不全导致的下肢肿胀、膝关节病变等鉴别；疑似PE的患者需要与心肺功能异常的各种疾病相鉴别，主要包括肺炎、胸膜炎、气胸、急性肺水肿、支气管扩张、心肌梗死、心肌炎及心包炎、心包填塞等。

四、MM相关VTE的风险因素

MM相关VTE通常在MM诊断后1年内（大多在诊断后6个月内）或MM复发时发生[6]–[7]。MM相关VTE风险因素见表3。其中，治疗相关因素显著增加了MM相关VTE的发生率，常见治疗药物的VTE相关风险见表4。无治疗相关因素时，MM相关VTE的发生率为3％～4％，单用IMiD、单用糖皮质激素或美法仑联合糖皮质激素（MP方案）不增加MM相关VTE的发生率，IMiD联合其他药物（如糖皮质激素或蒽环类为主的多药化疗）可使MM相关VTE发生率增高至10％～34％[6]–[8]。中国人群MM相关VTE的数据报道较少，有限的数据显示IMiD联合糖皮质激素在中国MM人群中的VTE发生率为3.3％～5.5％[9]–[11]。

**表3:** 多发性骨髓瘤（MM）相关静脉血栓栓塞症（VTE）的常见危险因素

患者因素
高龄
既往VTE病史
近期手术史
制动
合并其他基础疾病（肥胖、糖尿病、心肌梗死等）
疾病相关因素
高M蛋白血症
继发淀粉样变性（以肾病综合征为主要表现者）
治疗相关因素
免疫调节剂（沙利度胺、来那度胺、泊马度胺）
蒽环类为主的多药化疗
糖皮质激素
卡非佐米
促红细胞生成素
中心静脉置管

**表4:** 多发性骨髓瘤（MM）常用药物的静脉血栓栓塞症（VTE）相关风险

药物	VTE相关性
沙利度胺	单药不增加VTE风险，联合化疗或糖皮质激素显著增加VTE风险[7]
来那度胺	至少在复发患者中单药不增加VTE风险[12]，联合糖皮质激素显著增加VTE风险[13]–[14]
泊马度胺	数据有限，在大部分临床试验中已常规给予VTE预防处理[15]–[16]
蒽环类为主的多药化疗	蒽环类为主的多药化疗可增加VTE风险[6]
糖皮质激素	单药或联合美法仑时不增加VTE风险[17]–[18]，和IMiD合用时增加VTE风险（高剂量时更为显著）[13]–[14]
卡非佐米	与IMiD和糖皮质激素合用时，增加VTE风险[19]–[20]
硼替佐米	联合IMiD和糖皮质激素不增加VTE风险，甚至可能降低IMiD的VTE风险[21]
伊沙佐米	单药[22]–[23]或联合IMiD和糖皮质激素[24]–[25]不增加VTE风险
美法仑	单药或与糖皮质激合用时不增加VTE风险[17]–[18]
达雷妥尤单抗	单药或与其他药物合用时，不增加VTE的风险，甚至有降低VTE风险的倾向[26]–[27]
塞利尼索	目前尚无VTE相关性的报道

注：IMiD：免疫调节剂

五、MM相关VTE的预防

（一）MM相关VTE的分层预防策略

对于MM患者VTE的预防，国际骨髓瘤工作组（IMWG）[28]、欧洲骨髓瘤网络（EMN）[29]、美国国立综合癌症网络（NCCN）[30]等均推荐采用分层预防的策略。我们综合不同循证级别的研究及国外指南[30]–[34]，结合国内MM患者的临床特征和适用的治疗，拟定了中国MM患者VTE风险分层系统（表5），根据不同的风险分层采用阿司匹林、华法林、低分子肝素（low molecular weight heparin, LMWH）或直接口服抗凝剂（direct oral anticoagulants, DOAC）进行预防。由于VTE的风险在开始治疗后6个月内最高[6]–[7]，建议VTE预防至少6个月[6]。强调开始VTE预防时的基线评估及后续的动态监测。后续VTE预防与否以及使用何种预防策略需综合原发病治疗效果、后续治疗方案的选择、并发症和出血风险等因素进行评价。

**表5:** 多发性骨髓瘤（MM）相关静脉血栓栓塞症（VTE）风险分层及预防推荐[30]–[34]

因素	积分
个体和疾病因素	
遗传性易栓症[35]	6
VTE家族史	5
VTE病史	5
狼疮抗凝物阳性	4
骨盆、臀部或股骨骨折	4
浅静脉血栓病史	3
患者需要卧床超过72 h	2
大手术（1个月内）	2
抗心磷脂抗体阳性	2
年龄>75岁	2
年龄60~75岁	1
体质指数≥25 kg/m^2^	1
M蛋白浓度≥30 g/L[36]–[37]	1
充血性心力衰竭（1个月内）	1
急性心肌梗死	1
肺功能异常（慢性阻塞性肺疾病）	1
糖尿病	1
肾病综合征	1
治疗因素	
免疫调节剂	4
每周期地塞米松总量>160 mg	4
每周期地塞米松总量120～160 mg	2
蒽环类为主的多药化疗	3
卡非佐米[19]–[20]	2
中心静脉置管	2
促红细胞生成素	1

注：VTE风险分层及预防推荐：低于6分：不建议药物预防；低危组（6～8分）：阿司匹林；高危组（9～12分）：华法林（维持凝血酶原时间国际标准化比值2～3），预防剂量低分子肝素（LMWH）或直接口服抗凝剂（DOAC）；极高危组（≥13分）：治疗剂量LMWH或DOAC

阿司匹林：推荐用于MM相关VTE低危患者，推荐剂量100 mg/d，可有效预防VTE的形成。因为效果尚不明确，不推荐用于高危患者。

华法林：推荐用于MM相关VTE高危患者的预防，建议选择合适剂量的华法林维持维持凝血酶原时间国际标准化比值（INR）2.0～3.0[38]–[39]。

LMWH：推荐MM相关VTE高危患者采用预防剂量LMWH（如依诺肝素40 mg/d），极高危患者可采用治疗剂量LMWH（如依诺肝素1 mg/kg每日2次）进行VTE预防[33]。

DOAC：由于阿哌沙班目前在国内仅批准用于髋关节置换术后DVT预防，故推荐MM相关VTE高危患者采用预防剂量的利伐沙班10 mg/d，极高危患者可采用治疗剂量利伐沙班（20 mg/d）进行VTE预防[31]。

使用任何VTE预防药物都伴有出血风险，出血风险的评估应伴随整个血栓预防过程，MM患者出血风险的评估可参考表6[40]。当患者存在大出血或活动性出血等药物预防禁忌时，可考虑选用机械预防手段（如逐级加压弹力袜或间歇性充气加压）。待出血风险降低至可接受药物或消除时，若仍存在VTE的风险因素，可停止机械预防转为药物预防[41]。由于治疗药物的选择可以显著改变MM相关VTE的风险，对于高出血风险或有相关禁忌无法使用药物预防的患者，推荐首选硼替佐米、伊沙佐米及达雷妥尤单抗等无明显促血栓风险的药物组合，尽量避免IMiD、多药化疗及大剂量糖皮质激素为主的药物组合，以防止高VTE风险和高出血风险并存的临床困境。

**表6:** 多发性骨髓瘤（MM）出血风险评估

风险因素	积分
活动性消化道溃疡	4.5
3个月内出血史	4
血小板计数<50×10^9^/L	4
年龄≥85岁	3.5
肝功能不全（凝血酶原时间国际标准化比值>1.5）	2.5
重度肾功能不全（肾小球滤过率<30 ml/min/1.73 m^2^）	2.5
入住重症监护病房（ICU）	2.5
中心静脉置管	2
风湿性疾病	2
活动性肿瘤	2
年龄40～84岁	1.5
男性	1
中度肾功能不全（肾小球滤过率30～59 ml/min/1.73 m^2^）	1

注：<7分为低风险组，≥7分为高风险组

（二）伴肾功能不全和血小板减少患者的预防策略调整

MM患者常因疾病本身或治疗药物的不良反应出现肾功能不全、血小板减少。肾功能不全时使用阿司匹林可能加重肾脏病变，应慎用，可选择予以华法林治疗，并根据INR 2.0～3.0进行剂量调整。对于严重肾功能损伤（肾小球滤过率<30 ml/min/1.73m^2^）的患者，使用LMWH进行VTE预防时应考虑减量，有条件时进行抗Xa活性监测[42]，不推荐使用利伐沙班。血小板计数>50×10^9^/L的患者，因血小板减少导致的出血风险相对较低，若无出凝血指标异常或活动性出血，药物预防通常无需调整剂量。血小板计数<50×10^9^/L的患者，目前安全性数据有限，建议综合评估出血和VTE的风险后给予个体化治疗。

（三）MM相关VTE预防的疗程

对于长期使用IMiD、多药化疗、糖皮质激素等联合治疗的患者，建议持续VTE药物预防。与MM疾病本身及治疗无关的高危因素（如房颤、人工机械瓣膜置换术）持续存在时，应继续按照相关疾病的管理原则决定抗凝药物的使用。VTE的风险在开始治疗后6个月内最高[6]–[7]，6个月后，可综合原发病的治疗效果及治疗方案的改变进行调整。

无其他独立相关危险因素如房颤、人工机械瓣膜置换术以及其他个体因素（表1）时，MM原发病获得完全缓解（CR）或严格意义的完全缓解（sCR）的患者，巩固或维持治疗阶段若无IMiD及糖皮质激素使用，可停止VTE药物预防；MM原发病获得CR或sCR、巩固治疗阶段方案为IMiD联合糖皮质激素或化疗的患者，应继续VTE药物预防；MM原发病获得CR或sCR、维持治疗阶段单用IMiD的患者，可考虑停止VTE药物预防[6],[12]；MM原发病复发时，参考初治患者进行VTE药物预防。

六、MM相关VTE的治疗及随访

推荐参考肿瘤相关VTE的处理。

存在抗凝禁忌证的近端DVT或PE可考虑置入静脉滤器[43]。

无抗凝禁忌证的MM相关VTE应尽快启动抗凝治疗，可综合患者的并发症、个人意愿及药物安全性选择DOAC、LMWH或华法林。若采用利伐沙班，建议初始剂量15 mg每日2次，持续3周后改为20 mg/d [44]。若采用LMWH，依诺肝素可予1 mg/kg每日2次，持续1个月后改为1.5 mg·kg^−1^·d^−1^[45]；达肝素200 U·kg^−1^·d^−1^，1个月后改为150 U·kg^−1^·d^−1^[46]。若采用华法林，一般先应用LMWH至少5 d（依诺肝素1 mg/kg每日2次或达肝素200 U·kg^−1^·d^−1^[47]–[48]），至INR≥2.0再过渡到华法林单药，根据INR 2.0～3.0进行剂量调整。

抗凝治疗的疗程至少3～6个月。MM处于活动期时可综合评估血栓复发和出血风险后给予超过6个月的抗凝治疗。MM相关VTE复发的患者需要考虑长期抗凝治疗。

对伴有血流动力学异常的PE或大面积的DVT伴严重症状性肿胀或危及下肢的缺血患者，需同时启动导管或手术取栓或溶栓治疗以减少远期并发症[49]。常用的溶栓药物包括链激酶、尿激酶和新型重组组织型纤溶酶原激活剂（如阿替普酶）。

目前尚无研究探讨MM相关VTE发生后是否需要停用MM药物治疗，考虑到IMiD等MM治疗药物的促血栓风险，因此建议进行MM相关VTE抗凝治疗的同时谨慎使用或减量使用IMiD、蒽环类为主的多药化疗及大剂量糖皮质激素为主的药物组合，或考虑替换为硼替佐米、伊沙佐米、达雷妥尤单抗等无明显促血栓风险的药物组合。
